# “But Some People Still Think That Men Cannot be Raped”: A Qualitative Study on Portuguese Judges’ Perceptions Regarding Rape Perpetrated by Women Against Adult Men

**DOI:** 10.1080/19317611.2025.2509827

**Published:** 2025-05-24

**Authors:** Eunice Carmo, Daniel Cardoso, Nélio Brazão, Joana Carvalho

**Affiliations:** ^a^ Center for Psychology at University of Porto, Faculty of Psychology and Educational Sciences, University of Porto, Porto, Portugal; ^b^ Centre for Research in Applied Communication, Culture, and New Technologies, Lusófona University, Lisboa, Portugal; ^c^ School of Social Sciences and Humanities, NOVA University of Lisbon, Lisboa, Portugal; ^d^ Faculty of Psychology and Educational Sciences, CINEICC – Center for Research in Neuropsychology and Cognitive Behavioral Intervention, University of Coimbra, Coimbra, Portugal; ^e^ William James Center for Research, Department of Education and Psychology, University of Aveiro, Aveiro, Portugal

**Keywords:** Framework analysis, gender, judges, male rape myths, sexual violence

## Abstract

**Introduction:**

The perceptions of judges regarding sexual violence perpetrated by women against men (SVWM) have not been approached widely in previous empirical research. This exploratory qualitative study aimed to provide a preliminary understanding of the perceptions of Portuguese judges regarding SVWM.

**Method:**

Eight Portuguese judges (men and women) were interviewed in 2020. Data was analyzed using framework analysis.

**Results:**

Gender and alcohol had central roles in judges’ accounts, as non-consent, motivations, risk, the impact of violence, and sexual scripts were mostly discussed with these two factors in mind. Narratives oscillated between gender-neutral reflections and depictions of gender stereotypes and male rape myths. Challenges and opportunities of the Justice System were discussed considering the stigma associated with SVWM, while judges’ accounts were shaped by their lack of direct experience with such cases.

**Conclusions:**

Participants’ narratives reflected important contradictions between their adherence to some male rape myths and gender stereotypes and their endorsement of the ideal of a gender-neutral rape Law.

**Policy implications:**

The results of this study implicate that the impact of gender-based perceptions and rape myths on rape-related attrition rates and sentencing in SVWM cases should be further explored in empirical research. Additionally, public policy efforts should be invested in evidence-based professional training for judges focused on challenging gender stereotypes and male-rape myths.

## Sexual violence perpetrated by women against men

There has been a delay in the recognition of the sexual victimization of adult men by women both by research and by many legislative systems, including the North American, the British and the Portuguese ones (Fisher & Pina, 2013; Hlavka, 2017; Lowe & Rogers, 2017; Ralston, 2012; Rumney, 2007). However, prevalence data confirms that this victimization is frequent and thus needs to be addressed (Bullock & Beckson, 2011; Du Mont et al., 2013; Thomas & Kopel, 2023). Prevalence ratings of SVWM vary across studies and tend to be underestimated due to underreporting (Fisher & Pina, 2013; Walker et al., 2005). As an example, in an online survey of 260 Portuguese women studying in college, 35.8% of participants reported having committed some form of SV against men. Of those, 46.2% committed sexual coercion (e.g., threats and blackmail), 34.1% committed sexual abuse (e.g., use of power and authority) and 19.8% used physical violence (e.g., use of a weapon; Carvalho & Nobre, 2016). Moreover, in a sample of 1124 heterosexual British men, 71% had experienced some form of SV committed by a woman at least once during their lifetime (e.g., 39.8% had experienced attempted or completed forced vaginal or anal penetration; Madjlessi & Loughnan, 2024). In comparison, in Western countries, the prevalence of male-on-male rape or sexual assault is reported to be between 5 and 10% of all sexual assaults per year (Thomas & Kopel, 2023). Considering adult sexual assault, most studies report the mean age of male victim within a range of 20–30 years (Bullock & Beckson, 2011; Thomas & Kopel, 2023). Additionally, the sexual victimization of adult men by women has shown significant associations with depression, anxiety, and post-traumatic stress disorder, among other aversive impacts of SVWM (Madjlessi & Loughnan, 2024; Munroe & Shumway, 2022; Ralston, 2020; Walker et al., 2005; Weare, 2021).

## Social scripts regarding SVWM

The delay in the recognition of SVWM can be partly explained by social scripts, rape myths and gender stereotypes (Bates et al., 2019; Bates & Weare, 2020; Fisher & Pina, 2013; Hlavka, 2017). Social scripting theory argues that individuals use internalized narratives (i.e., scripts) when making sense of emotions, thoughts, and experiences as guides to appropriate behavior, namely regarding sexual interactions (Wiederman, 2005). Rape myths are stereotypical false beliefs about SV, including the characteristics of victims and offenders (Bates et al., 2019; Carroll et al., 2019; Turchik & Edwards, 2012).

In this context, the myth of female innocence is a foundation for the invisibility of women who commit sexual offenses, since it disavows that women can be perpetrators. This can be explained due to the significantly lower frequency of offenses committed by women, and due to gender stereotypes that de-sexualize women and attribute them nonviolent, passive, and caring roles (Bates et al., 2019; de Oliveira et al., 2024; Denov, 2003; Fisher & Pina, 2013; Geppert, 2022; Hayes & Carpenter, 2013; Wiederman, 2005). Social scripts also apply to men, usually assigning them an active role in sexual interactions, often associated with aggressive behavior, assumed to be incoherent with the possibility of sexual victimization (Bates & Weare, 2020; Carvalho & Brazão, 2021; de Oliveira et al., 2024; Depraetere et al., 2020; Javaid, 2015; 2017, 2018; Walfield, 2021). Thus, social scripts and rape myths assume the innocence of women and imply the invisibility of the victimization of men (Carroll et al., 2019; Carvalho & Brazão, 2021; Hayes & Carpenter, 2013; Hlavka, 2017).

## Justice system’s responses to SVWM

Previous research has hypothesized that women may be treated differently from men in the Criminal Justice System, namely due to The Chivalry (Nagel & Hagan, 1983; Steffensmeier et al., 2006). This hypothesis argues that women who commit particularly violent crimes (e.g., sex crimes) would benefit from a leniency effect, as their motives would not be interpreted as intentionally criminal or would be justified via coercion from a male partner or mental health problems, for instance (Beeby et al., 2021; Deering & Mellor, 2009; Steffensmeier et al., 2006). According to the Chivalry Hypothesis, this difference would be based on a paternalistic attitude toward women that would lead judicial professionals (namely judges) to approach them as less culpable and dangerous, and more worthy of protection (Embry & Lyons, 2012; Geppert, 2022; Nagel & Hagan, 1983; Sandler & Freeman, 2011).

This hypothesis has been tested by several studies looking at sex crimes committed by women. Studies found differences in sentence length for the same offenses across different typologies of sex offenses when comparing men and women (Embry & Lyons, 2012; Shields & Cochran, 2020). However, these results should be interpreted with caution, as other studies have only found this leniency effect in some conditions (e.g., when the crime was violent or when a weapon was used; Beeby et al., 2021; Dara Shaw et al., 2022; Sandler & Freeman, 2011). These results imply that a factor explaining different responses from the Justice System to women who commit sex offenses are the judicial professionals’ biased scripts and myths toward these crimes and the individuals who commit them.

A set of biased scripts that may be particularly relevant in SVWM cases are male rape myths. These include a) men cannot be raped; b) only gay men can be victimized or perpetrate SV; c) “real men” can defend themselves from rape; among others (Turchik & Edwards, 2012; Walfield, 2021; Widanaralalage et al., 2022). Male rape myths can have relevant impact when held by judicial professionals (such as judges) involved in decisions which may be crucial to the prevention of future crimes (Carvalho & Brazão, 2021; Walfield, 2021).

## Rape-related attrition

Attrition refers to the loss of cases within the criminal Justice System, particularly from the moment of a police report to conviction, focusing on the challenges of prosecuting a crime once it reaches the legal system (though it must be noted that sex crimes tend to be vastly underreported; Jehle, 2012; Pattavina et al., 2021; Sinclair, 2022; Spohn & Tellis, 2012; Taylor & Gassner, 2010). Rape-related attrition rates (attrition rates concerning sex crimes) indicate that such cases are often dismissed, lost, or withdrawn at some point between reporting and sentencing (Daly & Bouhours, 2010; Murphy-Oikonen et al., 2022; Sinclair, 2022). Studies of rape-related attrition indicate that this effect is pervasive regardless of the sex crime type and victim and offender characteristics (Hoffman et al., 2024).

Analyzing the impact of gender and rape myths on judges’ perceptions of sex crimes seems crucial to further understand rape-related attrition. On the one hand, traditional sexual scripts, gender stereotypes, and male rape myths can impact victimized men by increasing underreporting, rape-related attrition, re-victimization, and increased stigma in SVWM cases, especially when held by judges (Depraetere et al., 2020). On the other hand, judges’ biased social scripts about women who commit sex offenses (e.g., female innocence), can also hinder appropriate criminal investigations and prosecution, by creating obstacles to their rehabilitation and re-integration and increasing their levels of risk (Carvalho & Brazão, 2021; Denov, 2003; Gakhal & Brown, 2011).

## The Portuguese context

The delay in the recognition of sexual victimization of men by the Portuguese Law is illustrated by the fact that the first Penal Code applied since the installation of a democracy (dating from 1982) defined the victim of rape as a woman. The phrasing of the crime only became gender-neutral in 2007.

Portugal ratified the Istanbul Convention (Council of Europe Convention on preventing and combating violence against women and domestic violence) in 2013, which translated to significant legislative updates regarding sex crimes in 2015 (Sottomayor et al., 2015). For this reason, the Portuguese Law is a consent-based Law which does not specify the gender of the offender(s) or victim(s), in contrast with other legal systems (Fisher & Pina, 2013; Lowe & Rogers, 2017; Sottomayor et al., 2015).

## Current study

Exploring male rape myths and how the perceptions of judges may reflect the Chivalry Hypothesis in a sample of Portuguese judges^1^, who work with a gender-neutral consent-based Law, allows us to focus on what judicial professionals bring to the process. The aim of this exploratory study was to understand how judges perceived the gendered dynamics between the victim and the offender in a SVWM case, how they regarded the presumed (ir)relevance of any gender dimension to the case and its legal implications, and how they regarded the Justice System’s response to SV. Our study addresses perceptions regarding male rape myths to capture how these may influence judges’ perceptions and interactions with the Portuguese Justice System, regarding SVWM.

## Method

### Procedures

This study was conducted by senior and junior academics and clinicians, specializing in both qualitative and quantitative research, with diverse sociodemographic backgrounds.

The study was approved by the Ethics Committee of the Lusófona University. This study consisted of eight semi-structured interviews with Portuguese judges (women and men), conducted by the fourth author. The Portuguese Center for Judicial Studies was contacted by the research team and became responsible for inviting potential participants and providing the final list to the research team. So, judges were informed about the study, and those who were willing to participate expressed their availability. The team contacted 16 judges indicated by the Portuguese Center for Judicial Studies, of which eight agreed to participate in the study. For confidentiality reasons, the research team only had access to the names of the judges who accepted participation. Participants provided written informed consent before the interviews. Interviews took place at the judges’ offices between January and March 2020 and lasted an average of 45 minutes, ranging between 35 and 60 minutes. All interviews were conducted in European Portuguese and audio recorded. Participants were asked to read a vignette describing a sex crime committed by a young woman against a young man during a wedding (Appendix I). This was an original hypothetical vignette, adapted to the aims of this study and designed according to previous literature on SVWM, rape myths, and sexual scripts. This vignette was derived from, and associated with, a similarly-developed vignette successfully used in interviews with college-aged young people, as part of the same research project; the results of which have already been published (de Oliveira et al., 2024).

Then, the interview was conducted using an original semi-structured interview script (Appendix II) containing 25 questions, which served as the basis for data collection. Participants were asked about their professional training and experience; about their perceptions regarding how the judicial system handles SV cases; and about the vignette presented. The questions regarding the vignette were designed to address male rape myths.

### Data analysis

All interviews were transcribed verbatim in European Portuguese.

The data were analyzed using framework analysis (Ritchie & Spencer, 1994). Framework analysis is a qualitative method that is particularly suited to studies with pre-designed samples (such as groups of professionals), with a priori orientations (such as male rape myths) and using semi-structured interview guides (Gale et al., 2013; Ritchie & Spencer, 1994; Srivastava & Thomson, 2008). The main goal of framework analysis is to describe, analyze and interpret the characteristics of a particular phenomenon and to develop an analytical framework for the research study (Ritchie & Spencer, 1994). Besides, using framework analysis, authors can compare data between and within cases, using a systematic, flexible, and iterative process, combining deductive and inductive approaches (Gale et al., 2013). Thus, the research team believed that this tool would be the most adequate for our research question (Gale et al., 2013; Srivastava & Thomson, 2008).

This analysis consisted of five steps including familiarization, identification of a thematic framework, indexing, charting and mapping/interpretation (Srivastava & Thomson, 2008). In this case we sought to understand how judges perceived both the man and the woman in this case, how they regarded the putative relevance of any gender dimension to the events and their legal implications, and how they regarded the Justice System’s approach to SV. However, we did not establish *a priori* which issues would be brought up by the interviewees and still had to therefore follow all the stages associated with framework analysis.

In the familiarization stage, the first author read and re-read all eight transcripts, taking notes on preliminary analytic ideas, which were shared with all authors and discussed in order to gather an overview of collected data. In the second stage, the first author analyzed all the interviews and identified emerging themes and codes. A preliminary coding framework was developed at this stage and reviewed by all authors, using NVivo (Bazeley & Jackson, 2007; QSR International, 2022). These initial codes were revised by all authors, and changes were made when there were conflicting interpretations of what different excerpts could be coded for. In the indexing stage, all authors revised the coding and adapted it to a broad overview of the data. The charting and mapping/interpretation stages were operationalized using a framework matrix of all themes, subthemes, codes, and relevant illustrative quotes for all participants. This iterative process continued until all authors agreed on the coding structure, and its ability to comprehensively convey the results emerging from the data. The final framework structure was then revised by all authors. The authors take responsibility for the integrity of the data and the accuracy of the analyses.

## Results

### Participants

Participants of the current study included eight Portuguese judges: three (37.5%) men and five (62.5%) women, aged between 45 and 60 years old (*M* = 51.63, *SD* = 5.15). On average, participants had 17 years of experience serving as judges in SV cases, with experience ranging from 12 to 21 years (*M* = 17.12, *SD* = 2.10). In line with existing recommendations (Vasileiou et al., 2018), we note that our sample provided us with a situation in which at a certain point the respondents were reiterating existing talking points – that is to say, the last interviews conducted did not add any more nuance, nor did we manage to generate new codes or frameworks that were not already present in the analysis. Given the relative homogeneity of the target population, this was not unexpected, and it does not compromise the quality and reliability of the exploratory data presented below but rather constitutes a preliminary result in itself. Considering the limited pool of possible respondents and the exploratory nature of this work, we consider that we have been able to engage with the most salient narratives around our topic.

**Figure 1.**
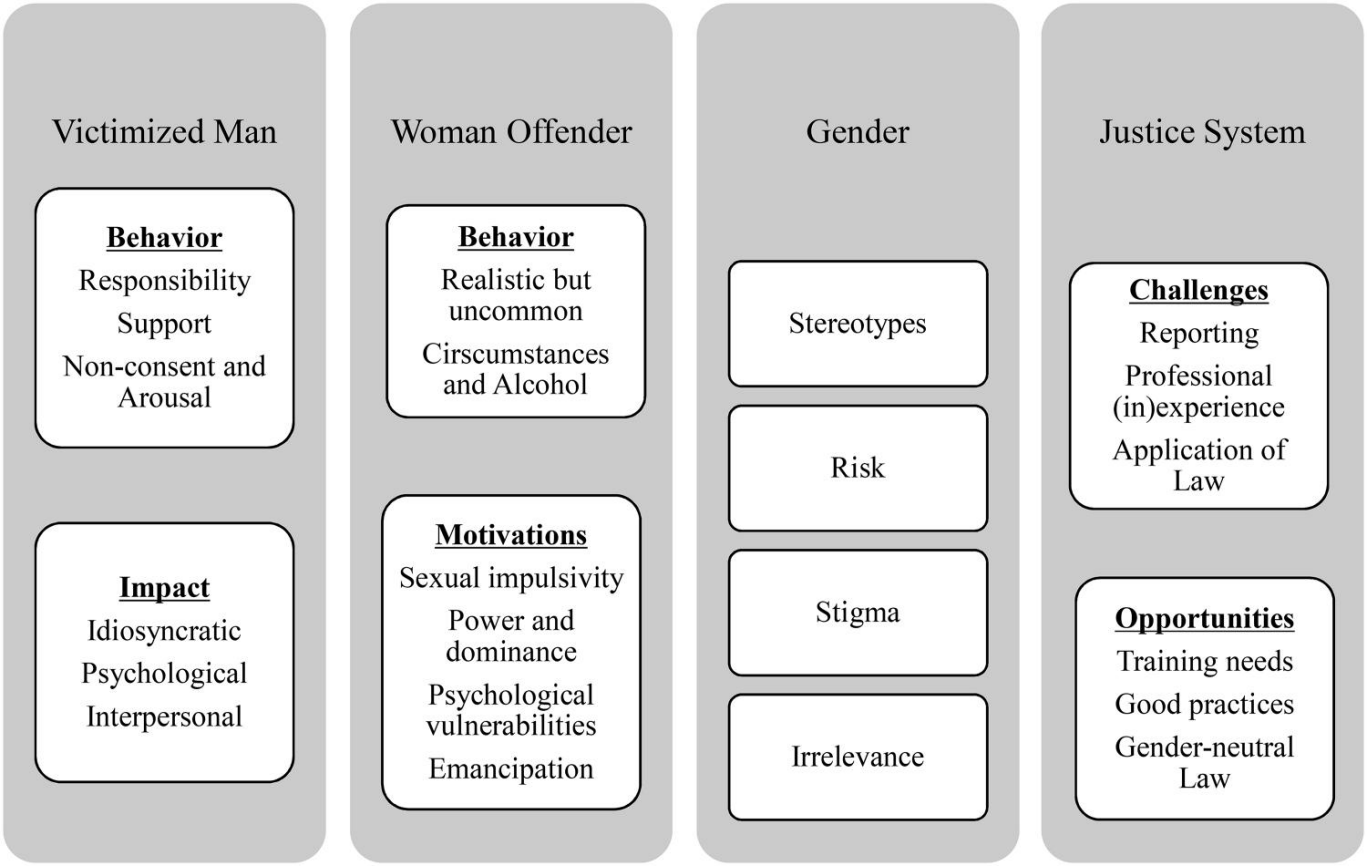
Analytical framework.

Four main themes were used to convey the meanings we surmised from the data, through framework analysis: 1) Victimized Man (with subthemes: behavior and impact); 2) Woman Offender (with subthemes: behavior and motivations; 3) Gender (with codes: stereotypes; risk; stigma; and irrelevance); 4) Justice System (with subthemes: challenges and opportunities). See Figure 1 for a graphic representation of themes, subthemes, and codes.

### Victimized Man

#### Behavior

Regarding the victim’s behavior, two judges attributed some personal *responsibility* to the victimized man, as expressed here:

He may not have had the conditions to stop her from entering the room. He could have been very nauseous and maybe let her in because he needed help. But if that was not the case, he should not have allowed her in the room. (…) I think he could have had a different preventative behavior (P4)

In contrast, most judges *supported* the victim’s reaction and expressed emphatically that he could not have done anything differently (P5: *“I do not see what else he could have done, especially considering the state he was in.”*). Here, an association with the code “circumstances and alcohol” becomes clear, as many attributed the victim’s inability or unwillingness to consent to alcohol. They also added that his previous behavior was not indicative of consent and should not shape any considerations regarding the violent nature of the situation.

Some participants argued for the inexistence of an association between physiological sexual arousal (in this case, an erection) and sexual consent (P1: *“I want to make this clear: The fact that he got an erection does not mean that he gave his consent”*). These participants argued that this lack of an association was both present in men and in women. In contrast, other judges were less certain on this topic (P4: *“I do not know what kind of physical reaction a man has to unwanted sexual stimulation from a woman. I think alcohol may have disinhibited him and, despite not wanting it, being able to have an erection”*).

As illustrated by the last quote, the subtheme behavior is intricately connected with the code regarding circumstances and alcohol, where participants reflected on the importance of these factors for a judgment regarding this case.

#### Impact

Concerning the impact of the abuse, participants believed it would be *idiosyncratic,* as expressed here:

It could lead, on the one hand, depending on the person, to someone to think «I did not want this, but I am glad that even while drunk I could do it»; or, on the other hand, he could have felt used against his will, sexually abused and that might have caused suffering. (P1)

Nonetheless, different types of impact were mentioned, namely psychological and interpersonal impacts. Additionally, the subtheme impact has a direct link with the code “gender irrelevance”, as many participants believed gender was irrelevant when considering the impact of SV and that the personal idiosyncrasies of each individual would be more relevant.

### Woman offender

#### Behavior

When reflecting on the woman’s behavior, most participants considered it realistic but uncommon and expressed their belief that SV perpetrated by men may seem more realistic and frequent for several reasons (P6: *“I believe it probably is not known because it is not reported, not because it does not happen. I think it is realistic that it would happen. From my standpoint as a judge, this is not frequent in courts”*). Importantly, some judges reflected on the impact of gender on their answers (P5: *“I believe the situation is possible, without a doubt. I do not know if it occurs frequently, namely due to gender issues.”*) This code associates with the code regarding reporting and the code regarding professional (in)experience, as many judges were aware of the tendency for underreporting in SVWM and explicitly based many of their answers on their lack of direct experience.

Judges also analyzed the woman’s behavior considering the circumstances and the consumption of alcohol. Most participants considered alcohol a crucial factor to consider from a juridical point of view (P1: “*Alcoholic intoxication is relevant because it can impact willingness and arousal and the physical ability to react”*). Many believed this sort of abuse would be more likely in social situations involving alcohol. Additionally, one participant argued that alcohol could have been relevant to understand the offender’s behavior (P8:*” I think the alcoholic intoxication may have disinhibited and provided more courage to Mary”*). Finally, one participant considered the alcoholic intoxication a necessary condition for a similar case of SVWM to occur (P5: *“I believe this situation always assumes the intoxication (…) as the case does not describe any physical violence.”*). Nonetheless, some participants acknowledged that this sort of abuse could have happened in other contexts and circumstances, such as with an established couple.

#### Motivations

Many participants mentioned sexual impulsivity as a motivation (P1: *“a huge sexual desire that grew during the night and the expectation to achieve it, and then the inability or lack of willingness to dominate that desire*”). One participant shared that this would contrast with their gender stereotypes regarding the sexuality of women:

It may also be associated with her personal characteristics, which I do not believe are common or trivial in women, but she might have had an increased stimulus for sexuality. (…) Because it is not very normal that a woman has a quick orgasm following penetration (P8).

Many participants also attributed the violent behavior to a need for power and dominance (P1: “*I think it could be something more complex like the desire to dominate* [and] *subjugate the man”*). Most of these justifications did not take gender into account.

Two participants mentioned psychological vulnerabilities, without considering gender, pathologizing the offender.

Finally, two participants also argued that the emancipation of women, regarding their sexuality, might have contributed to the situation (P4: *“Women became more autonomous, more aware that they are equal to men, namely at the sexual level (…) This had positive and negative consequences. In this case it was a negative consequence”*).

### Gender

#### Stereotypes

This code was used when participants reflected upon and challenged gender stereotypes and male rape myths, as expressed here:

Many people still think that men cannot be raped. But they can be raped. They assume that men are always ready to have sexual relationships. But this is a stereotype that cannot be accepted. (P4)

The code was also used when participants shared ideas that were coherent with current gender stereotypes and (male) rape myths, namely sharing common sexual scripts (P5: *“While not following fixed rules, I would say that it is more common for men to take initiative in the beginning of sexual relationships*”).

These stereotypes are applied to forensic matters (P6: “*Men are more likely to commit crimes generally. I believe I am not being prejudiced. I do not think this is due to cultural reasons, but to biological and genetic ones, maybe also to cultural ones”*).

#### Risk

Considering gendered risk, some participants thought that men who commit sex offenses would be more dangerous, perceiving SV to be fundamentally a physical crime, and focusing mostly on men’s physical strength (P2: *“In terms of the ability to physically endanger someone, I believe the man who sexually offends would be more dangerous, considering his physical force and his ability to injure the body*”). This also led some participants to believe that, barring the premeditated use of intoxicants by the woman, men’s average superior physical strength makes SV by women less likely.

Another judge associated the level of risk with gendered motivations for SV:

I would say that in men the dangerousness is associated with uncontrollable compulsive behaviors, eventually a psychopathological profile. In women, I would associate these behaviors with a sociocultural issue, of power, not so much with the sexual impulse that she cannot control. (P7)

However, not all agreed, though one participant understood risk via harm: “*I would tend to say that they are equally dangerous because the effects (…) will have a similar damaging impact*”).

#### Stigma

The code stigma describes situations in which participants mentioned the invisibility of men’s victimization (P7: “*One day we will have a male #MeToo, of the men who were sexually abused and who currently are not aware of that (…) are ashamed of not having been able to do something about it”*), and of the stigma surrounding it as an added risk to the victim brought about by seeking legal recourse.

#### Gender irrelevance

The code gender irrelevance refers to statements where participants describe believing that the gender of the individual does not influence their perceptions (P2: *“I don’t find any motivations for men that I don’t also acknowledge for women. I think the motivations would be the same.”*). This code was applied in situations where judges described the situation in the vignette, the offender’s motivations, the risk of sex offenders, and other broader accounts, as long as it included a disavowing of gender as being relevant to the interpretation of what was being said (P6: *“That idea is based on a narrative of a different sexuality in men and in women, but that does not exist. It does not apply to sexuality nor to human behavior”*).

### Justice system

#### Challenges

Participants identified barriers to the reporting of SVWM, such as the ones described here:

I think it is harder for a man to make a report like this than for a woman. But it is hard for both. Many sexual crimes never reach the authorities because victims are ashamed (…) because there is still a social narrative that men cannot be victimized by women, although things have been evolving (P4).

One participant considered that the main barrier faced by men existed prior to the reporting stage:

I do not think that the treatment of the victim after the communication to the Justice System would be different according to gender. I think the social treatment of the individual is different according to gender prior to that communication. (P6)

This code had a clear association with stigma, as judges associated the difficulties that victimized men face in reporting with shame and embarrassment. They also underlined that cases of child sexual abuse perpetrated by women may comparatively reach the courts more often precisely due to these barriers faced by victimized men (P3: *“I think that for children the reporting and complaint process is easier. For adults, there is more stigma”*).

Importantly, judges reported professional inexperience with situations of SVWM. Only one participant had had direct contact with child sexual abuse perpetrated by women. This was frequently brought up by participants as a way of justifying their answers.

Several participants mentioned the difficulty in applying the Portuguese Law, namely the consent-based description of rape, as mentioned here:

I think our Law is not as linear as the Istanbul Convention, which demands the absence of consent. Remembering the legal description of our Law, it says “whoever constraints someone else”. It does not say “whoever practices sexual acts without consent”, it does not say “without consent”, it says “constraints” which is a very ambiguous term which will cause controversy (P5).

Several judges were concerned about the issue of proving a sex crime when physical violence is not involved, since it potentially dematerializes consent and the lack thereof.

#### Opportunities

Respondents could not identify specific good practice guidelines for dealing with SVWM but mentioned general guidelines. Many judges mentioned “declarações para memória futura”, which is a judicial proceeding that allows the person who was victimized to be heard at the first stage of the judicial process, when the evidence is being gathered, without having to repeat the testimony at later stages. Participants also mentioned general best practice guidelines, including: the preservation of evidence; forensic medical examinations; respect for the victims’ privacy and integrity; the provision of specialized psychosocial support for victims; the physical separation of victims and offenders during judicial proceedings; and the need to ensure the use of standardized procedures across the country.

Several participants referred different *training needs* that would need to be targeted to improve the judicial proceedings in SVWM cases, in line with the aforementioned best practice guidelines (P6: *“We do not have training on how to interview and talk to people, without forgetting that we are not psychologists”*).

Respondents also underlined the gender-neutral approach of the Portuguese Law as an opportunity to improve best practices (P2: *“It is not violence perpetrated by a woman against a man or a man against a woman. It is independent of the gender of the person who offends”*).

## Discussion

This exploratory study was the first to provide an understanding of the perceptions of Portuguese judges regarding how gender is manifested or not in SVWM cases, and its interactions with the Justice System. Considering the influence of these perceptions on rape-related attrition rates (Daly & Bouhours, 2010; Murphy-Oikonen et al., 2022) these findings have great relevance for the understanding of how the Justice System deals with SVWM. The findings of this study can be understood through a framework composed of four themes: Victimized Man; Woman Offender; Gender; and Justice System.

In theme one, Victimized Man, most judges removed any responsibility from the victimized man, whilst others thought he could have done more to prevent the crime. However, even when some preventative responsibility was placed on the man, the judges did not underestimate the seriousness of SVWM. As highlighted in previous literature, professional training of judges should ensure that any victim-blaming narratives are contradicted, and that the onus of responsibility is always placed on the offender (Fuchs, 2004).

Judges described the man’s expressions of non-consent, and most argued that physical signs of sexual arousal were not indicative of consent, which indicates that most participants were informed on the available scientific evidence on this issue (Bullock & Beckson, 2011; Fuchs, 2004). Additionally, judges provided detailed explanations of their reasoning, which may highlight an awareness that this is a commonly misunderstood topic. Thus, this novel result contradicts some evidence from judicial studies which show that erections and ejaculations have often been interpreted as signs of consent by judicial professionals (Bullock & Beckson, 2011; Fuchs, 2004).

Participants provided several hypotheses regarding the impact of violence on the man, especially on a psychological and interpersonal level, while underlining that it would depend more on each person’s circumstances and resilience than on gender. Still, a participant considered the hypothesis that a man might have interpreted such a situation as a victory, in an example of a common male rape myth, previously reported in community samples (Depraetere et al., 2020; White & Kurpius, 2002; Widanaralalage et al., 2022). When mentioning the psychological impact of victimization, a few participants mentioned self-blaming attitudes, which shows understanding of victimization processes (Thomas & Kopel, 2023; Walker et al., 2005; Widanaralalage et al., 2022). Participants did not mention the physical impact of victimization, which may be less obvious when the person victimized is an adult man and the perpetrator is a woman (Du Mont et al., 2013).

Theme two, Woman Offender, reflected participants’ views on the behavior of the woman, considering the situation in the vignette a realistic but uncommon crime, while anchoring their reasoning on gender perceptions, available statistics, and lack of direct experience, in line with previous studies (Gakhal & Brown, 2011; Mellor & Deering, 2010; Rumney, 2007). While discussing the woman’s behavior, the circumstances and the role of alcohol were crucial. Some judges used alcohol as a justification for the woman’s behavior and all professionals considered it a crucial factor for the juridical decision, with one considering that alcohol was a necessary assumption of such a crime, contradicting the available literature on SVWM (Monk-Turner & Light, 2010). Additionally, judges provided examples of other contexts where similar crimes could have occurred (e.g., in the context of a hierarchical or romantic relationship), showing knowledge of the reality of SVWM (Carvalho et al., 2021). These results are in line with previous literature that showed that in cases of SV perpetrated by women, judicial professionals tend to pay closer attention to circumstantial and mitigating factors (Beeby et al., 2021; Deering & Mellor, 2009; Shields & Cochran, 2020). Our results seem to reveal that, in the judges’ perceptions, alcohol may have served three purposes: to invalidate the victimized man’s consent; to provide additional evidence of non-consent (inhibiting defensive behavior); and to disinhibit the women offender’s behavior.

Most judges identified sexual impulsivity as the main motivation for the crime. This is in line with a stereotypical idea that associates SV with out-of-control sexual desire and not with violent behavior; it is also in line with existing literature that shows that women perpetrators’ behaviors tend to be seen as romantic or promiscuous, instead of aggressive or violent (Cain & Anderson, 2016; Carroll et al., 2019; Clements et al., 2014; Gakhal & Brown, 2011; Struckman-Johnson & Struckman-Johnson, 1993). Furthermore, that interpretation runs counter to existing explanatory models of SV perpetrated by women (DeCou et al., 2015; Elliott et al., 2010). When describing sexual impulsivity, judges resorted to gender stereotypes to justify their answers. In contrast, the motivations associated with power and dominance and with psychological vulnerabilities were not described in a gendered framework. All the motivations mentioned are in line with some of the known etiological factors that help explain SVWM (Carvalho et al., 2021; Carvalho & Nobre, 2016; DeCou et al., 2015; Elliott et al., 2010). For instance, in a study conducted with a sample of Portuguese university students, sexually aggressive women were found to show higher levels of sociosexuality, sexual compulsivity, sexual excitation, sexual fantasies of dominance and submission, among other factors (Carvalho & Nobre, 2016).

Within theme three, Gender, judges expressed traditional sexual scripts and male rape myths, and in some cases applied them to forensic contexts, while also being able to challenge these scripts. This ambiguity was a steppingstone of the interviews. Most judges justified the increased risk of SV in men in comparison to women with the supposed differences in physical strength. Hence, they adhered to a central component of the Chivalry Hypothesis: they assumed women to be less culpable and dangerous, and more worthy of the protection of the Justice System (Embry & Lyons, 2012; Geppert, 2022; Nagel & Hagan, 1983; Sandler & Freeman, 2011). Previous research indeed shows that professionals tend to consider sexual offenses committed by women as less harmful and serious than those committed by men (Clements et al., 2014; Gakhal & Brown, 2011; Mackelprang & Becker, 2017; Mellor & Deering, 2010).

Participants also highlighted the central role of intoxication in this case. This may reflect an incomplete view of SV, placing the emphasis on hands-on forms of SV, including the use of physical force, while neglecting hands-off strategies. In contrast, recent evidence has shown that women who commit sexual offenses then to resort to a variety of strategies, both hands-on and hands-off (Carvalho & Nobre, 2016; Depraetere et al., 2020).

Additionally, some judges also justified gendered beliefs about risk with perceived differences in the origins of the offending behaviors, assuming that women offend mostly due to cultural reasons and men to biological and genetic ones. There is no scientific evidence to back up this claim, which mirrors the longstanding Nature vs Nurture debate (DeCou et al., 2015; Elliott et al., 2010). Additionally, this is another example of an interpretation of the woman’s behavior as not necessarily criminal but justified by circumstantial or sociocultural reasons, in line with the Chivalry Hypothesis (Beeby et al., 2021; Steffensmeier et al., 2006). These results are particularly important considering the literature on rape-related attrition. If judges minimize the risk posed by women who sexually offend, they are less likely to prosecute them, thus hindering opportunities for rehabilitation and potentially increasing the impact on victims (Beeby et al., 2021). Additionally, participants showed awareness regarding the increased stigma faced by men sexually victimized by women. This is supported by the current scientific literature on the impact of SVWM (Javaid, 2018; Ralston, 2012, 2020; Thomas & Kopel, 2023).

Importantly, judges often underlined that their perspectives were gender-neutral, placing emphasis on individual characteristics and on the Law. Thus, judges alternated between providing gender neutral accounts and resorting to gender stereotypes to back up their answers – this seeming contradiction, which went unremarked by participants, is a good example of how scripts can be incorporated into personal narratives seamlessly, facilitating the continued use of stereotypes, even in the context of legal work.

Finally, within theme four, judges reflected on the Justice System, mentioning challenges and opportunities. Participants’ acknowledgment of increased barriers faced by adult men, associated with stigma and lack of trust in the Justice System, is aligned with literature that shows that most SVWM crimes are not reported (Lowe & Rogers, 2017; Thomas & Kopel, 2023; Walker et al., 2005). A participant argued, in line with existing literature (Pattavina et al., 2021; Spohn & Tellis, 2012; Taylor & Gassner, 2010) that most of these barriers exist before the first contact with the judicial system. However, this also reflects the idea that the Justice System would provide an ideal treatment to victims regardless of their gender, which is incoherent with the judges’ narratives regarding the gender-specific challenges and stigma faced by victimized men (and women); and incoherent with reports of rape-related attrition caused by the withdrawal of police reports by victims (usually due to the negative experiences within the Justice System; Murphy-Oikonen et al., 2022; Thomas & Kopel, 2023; Walker et al., 2005).

Participants had no personal or professional contact with SVWM, which shaped many of the judges’ answers and stands in contrast with the current prevalence data on SVWM. This result is likely to be a consequence of rape-related attrition rates (Daly & Bouhours, 2010; Murphy-Oikonen et al., 2022; Sinclair, 2022). A recent qualitative study showed that police officers often prioritize rape cases that they predict to be most likely to reach a conviction, a decision that is influenced by, amongst other factors, rape myths and victim blaming attitudes (Sinclair, 2022). Thus, SVWM cases may not reach judges due to the impact of male rape myths on rape-related attrition in earlier phases of the judicial process.

Despite the consent-based Law’s merits, judges highlighted the difficulty in producing reliable evidence, a problem which they believed has not been ameliorated by the recent legislative updates (Merdović et al., 2022), illustrating another side to rape-related attrition, as these legislative updates may not be sufficient to overcome the difficulties in prosecuting such crimes (Daly & Bouhours, 2010; Murphy-Oikonen et al., 2022; Sinclair, 2022). Judges highlighted that the Justice System may not be ready to deal with all forms of SVWM, especially because of rape myths and social scripts (Carvalho & Brazão, 2021; Denov, 2003; Gakhal & Brown, 2011), as illustrated by victim’s accounts in previous studies (Murphy-Oikonen et al., 2022).

As for opportunities, when asked about specific SV good practice guidelines, participants could only mention general ones, most in line with those offered by men victimized by SVWM in previous research (Walker et al., 2005). This highlights the absence of specific protocols for dealing with SV cases, but also an awareness of such absence, and a broad understanding of the main pitfalls.

When describing the gender-neutral approach of the Portuguese Law, participants also reflected on the rape myths that often hinder its application. Despite its neutral formulation, the cases that the Law tends to reach are cases of SV perpetrated by men against women. This reflection is important considering the findings that show a gender gap in the prosecution of sex crimes (in the U.S.A.), in line with the Chivalry Hypothesis (Embry & Lyons, 2012; Sandler & Freeman, 2011; Shields & Cochran, 2020). The judges in this study argued in favor of maintaining the neutrality of the Law and prosecuting cases fairly, regardless of gender, but the reality of the Justice System may not reflect this aim.

## Limitations

This study is not without its limitations. Firstly, self-selection bias cannot be excluded. Judges that decided to take part in this study might have been more aware of SVWM or interested in the topic than other judges. Similarly, a social desirability effect might have occurred. The study did not attempt to conduct a direct comparison between judges who had experience with SVWM versus those who did not have that experience and therefore cannot consider what impact that experience might have on the results. Thus, the results of this study might overemphasize judge’s awareness of this phenomenon. Secondly, the methodology employed in this study does not guarantee high ecological validity. The perceptions reported were based on a hypothetical vignette, adapted to the aims of this study, so judges might have answered differently if they had been presented with a real case. During the interviews, participants did not have to consider a series of factors that shape their decision-making in real situations, such as heavy workloads, time constraints, or the influence of media attention to particular cases. Thus, the decision-making processes described by these eight judges would not necessarily be applicable in real judicial settings. Future studies should test this approach. Thirdly, as this was an exploratory study focusing on SVWM and gender, it did not take into account sexual preference, so future studies could benefit from considering this layer of analysis. Finally, a sample of eight judges is not ideal to ensure representativeness and diversity, namely concerning socioeconomic status, ethnicity, sexual orientation, religion, ability, and other factors. However, diversity was one of the factors accounted for during recruitment and was ensured as much as possible within the constraints of feasibility. Future studies with larger sample sizes and accounting for diversity could investigate gender differences in judges’ accounts, as those were not captured by this study.

## Public policy implications

Considering the exploratory nature of this study, the recommendations that follow aim to provide some guidance for potential studies to be able to draw methodologically-sound generalizations. Our research can be expanded by taking advantage of quantitative methods to evaluate whether the gender gap in the sentencing of sex crimes can be observed outside the U.S.A. (Embry & Lyons, 2012; Sandler & Freeman, 2011; Shields & Cochran, 2020). This would have important public policy implications, as further preventative measures could be taken to reduce the impact of gender-based perceptions and rape myths on rape-related attrition rates and sentencing in SVWM cases.

The specific educational and training needs of judicial professionals regarding SVWM could be further explored to design up-to-date evidence-based training programs, as identified by participants. Additionally, clearer good practice guidelines, adapted to men victimized by SVWM, should be in place across all points of contact between victims and the Justice System (Dara Shaw et al., 2022; Fuchs, 2004).

Considering the perceptions of the judges regarding difficulties in reporting, these results could also be further explored with groups of professionals who contact with people who suffered violence before they reach the Judicial System, such as police officers and health care professionals (Fávero et al., 2022; Fehler-Cabral et al., 2011; Kassing & Prieto, 2003; Sleath & Bull, 2017). Preliminary research shows that when these first responders endorse male rape myths, their initial contact with victims may be inadequate, contributing to stigma. In turn, the obstacles to reporting increase, which may then lead to greater rape-related attrition rates (Bates & Weare, 2020; Fávero et al., 2022; Javaid, 2016; Mellor & Deering, 2010; Thomas & Kopel, 2023). Public policy updates may be needed to tackle this issue, namely through professional training.

Future research could include studies regarding the recent consent-based Laws applied within the context of the Istanbul Convention, as this was described as a considerable challenge by our participants (GREVIO, 2019). According to them, these Laws have great merits, but difficulties in gathering evidence and prosecuting cases remain. Understanding these obstacles in greater detail is essential to tackle the issue of rape-related attrition rates. Further updating legislation globally to align it with the Istanbul Convention’s recommendations requires an international public policy effort, which should involve all relevant stakeholders (Depraetere et al., 2020).

## Conclusions

In conclusion, judges shared important contradictions between an ideally gender-neutral narrative about SVWM and the Law, and idiosyncratic ideas about motivations, risk and the sexual characteristics of men and women, which were often (but not always) aligned with gender stereotypes, sexual scripts, and rape myths. It is important to note that the judges’ ostensive gender-neutral approach did create a series of internal contradictions that the participants themselves could not or did not fully address, vis a vis their own lack of experience with (or knowledge of) cases of SVWM. While this gender-neutral approach is seen as positive, the redeployment of certain stereotypes about the motivations behind SV, and the disavowal of any gender-specific dynamics of SV, might contribute to the invisibility of SVWM. Additionally, our data partially supports a leniency effect toward women sex offenders, in line with the Chivalry Hypothesis (Beeby et al., 2021; Dara Shaw et al., 2022; Embry & Lyons, 2012; Sandler & Freeman, 2011; Shields & Cochran, 2020). Hence, judges can elaborate on a judicial response to rape that includes the hypothesis of men as victims and women as offenders, despite their social scripts, but not systematically so. These results highlight the importance of providing professional training that allows judicial professionals to challenge gender stereotypes and rape myths, in order to reduce rape-related attrition rates.

## Supplementary Material

Supplemental material Appendices.docx

## Note

1 In Portugal, judges’ training in the Centre for Judicial Studies is organized in three cycles of increased specialization, including theoretical and practical supervised training, as described here: https://cej.justica.gov.pt/ENGLISH/Initial-training. For a characterization of Portuguese judges, please consult Ferreira et al. (2014).
