# Supplementary material for: “But Some People Still Think That Men Cannot be Raped”: A Qualitative Study on Portuguese Judges’ Perceptions Regarding Rape Perpetrated by Women Against Adult Men
Source: Int J Sex Health. 2025 May 24;37(4):559–74. doi: 10.1080/19317611.2025.2509827 (PMC12867448; doi:10.1080/19317611.2025.2509827)
Supplement: Supplemental material Appendices.docx [file WIJS_A_2509827_SM6333.docx]

**Appendix I - Vignette**

Richard (man) and Mary (woman), both 33 years old, met at a wedding of mutual friends. During the wedding, Richard and Mary spent most of the time chatting and dancing together. Richard had several alcoholic drinks and, as the party progressed, got more intoxicated. Mary suggested that she could take Richard to his hotel room. When they got to the room, Richard threw himself on the bed. Mary helped him undress and bit his ear. She said she really wanted to “be with him”. Richard asked her to stop because he had drunk a lot, and he was not feeling well. Mary kept grabbing and groping him. Richard asked Mary to stop again because he felt undisposed. However, Mary opened his boxers, grabbed his penis, and told him “I want it now”. Immediately, Mary started stimulating Richard’s penis through oral sex, although he asked her to stop. As soon as he was erect, Mary went on top of him and had a quick orgasm. Richard could not believe he had had an orgasm and ejaculated. He felt confused and humiliated. After the incident, Mary gave Richard a goodbye kiss and left the room.

**Appendix II – Interview guide**

Part 1 – Male rape myths

1. Could you summarize the situation described in the vignette with your own words?
2. Do you think this situation is realistic? Why?
3. If the situation was described the other way around (i.e., if Richard was the one initiating sexual contact, and not Mary), would it seem more realistic? Why?
4. Do you think the situation described in the vignette happens frequently? Do you think it could happen in other contexts? [If yes] Which ones?
5. Do you think women often display the behaviors described in the vignette? Why?
6. In your perspective, why did Mary behave like that?
7. Do you think Mary’s behavior is reprehensible? How?
8. What do you think about Richard’s behavior? Do you think he could have said or done anything differently? [If yes] What?
9. Do you think Richard’s behavior is reprehensible? How?
10. Do you think Richard is in any way responsible for what happened? [If yes] How?
11. Do you think the situation described in the vignette could have any negative consequence for Richard? [If yes] How? [If not] Why?
12. Do you believe the situation in the vignette describes a sexual crime? [If yes] Which one?
13. [If a sexual crime is identified] Could you define [the sexual crime mentioned by the participant] using your own words? Why do you think the situation in the vignette describes a [the sexual crime mentioned by the participant]?
14. Is there anything else you would like to add?

Part II – Academic/Professional training and experience

1. What is your academic training?
2. Do you remember ever having learned about sexual violence during your academic studies? In which curricular units?
3. Outside your academic studies, have you ever searched for or took part in training regarding sexual violence?
4. [If yes] Was this training useful? In what way?
5. [If yes] In that training, was mentioned?
6. Within your professional occupation, is female-perpetrated sexual violence against men frequent?
7. [If not] Why do you think it is not frequent?
8. How does the justice system handle this issue?
9. Which good practice guidelines do you identify in the way the justice system deals with this issue?
10. Do you identify limitations or problems in the way the justice system deals with this issue? [if yes] Which ones?
11. In your perspective, what would be the motivations for a woman to sexually abuse a man? Do you think this woman can represent a danger to society? [If yes] In what way? Do you believe that women who commit sex offences pose the same level of dangerousness as men who commit the same crimes? Why?

[Debriefing]
